# Simultaneous synthesis of graphite-like and amorphous carbon materials via solution plasma and their evaluation as additive materials for cathode in Li–O_2_ battery

**DOI:** 10.1038/s41598-021-85392-2

**Published:** 2021-03-18

**Authors:** Chayanaphat Chokradjaroen, Hiroko Watanabe, Takahiro Ishii, Takahiro Ishizaki

**Affiliations:** 1 SIT Research Laboratories, Shibaura Institute of Technology, Tokyo, 135-8548 Japan; 2 Materials Science and Engineering, Graduate School of Engineering and Science, Shibaura Institute of Technology, Tokyo, 135-8548 Japan; 3 Department of Materials Science and Engineering, College of Engineering, Shibaura Institute of Technology, Tokyo, 135-8548 Japan

**Keywords:** Materials for energy and catalysis, Materials chemistry

## Abstract

Cathode materials are essential for enhancing electrocatalytic activity in energy-conversion devices. Carbon is one of the most suitable cathodic materials for Li–O_2_ batteries owing to its chemical and thermal stability. Carbon materials synthesized from tributyl borate (TBB) using a nonthermal solution plasma method were characterized using x‐ray diffraction, Raman, field emission scanning electron microscopy (FE-SEM), transmission electron microscopy, and x-ray photoelectron spectroscopy and were evaluated as additive materials for cathodes in a Li–O_2_ battery. Two separate carbon materials were formed at the same time, a carbon dispersed in solution and a carbon precipitate at the bottom of the reactor, which had amorphous and graphite-like structures, respectively. The amorphous carbon contained boron and tungsten carbide, and the graphite-like carbon had more defects and electronic conductivity. The crystallinity and density of defects in the graphite-like carbon could be tuned by changing the SP operating frequency. The Li–O_2_ battery with the amorphous carbon containing boron and tungsten carbide was found to have a high capacity, while the one with the graphite-like carbon showed an affinity for the formation of Li_2_O_2_, which is the desired discharge product, and exhibited high cycling performance.

## Introduction

The search for cathodic materials or additive materials that can efficiently catalyze the oxygen reduction reaction (ORR), for application in energy conversion devices such as Li–O_2_ batteries, has attracted considerable research interest in the last decade. Carbon-based catalysts have drawn attention because carbon materials are cost-effective and exhibit relatively high chemical stability^1^. Moreover, carbon itself is relatively active for the formation of the desired products generated from ORR during discharging in Li–O_2_ cells^2^. The desired products, such as lithium peroxide (Li_2_O_2_), usually occur on the cathode surface in discharged cells^3,4^. Normally, Li_2_O_2_ can decompose back into lithium ions (Li^+^) and oxygen (O_2_), which are reused for further ORR^5^. However, some unwanted products, such as lithium carbonates (Li_2_CO_3_) and lithium carboxylates (LiCO_2_R; R is hydrogen or alkyl), are also generated because of the instability of the Li_2_O_2_ species^2^. One of the parameters that influence the formation of discharge products is the surface and bulk structures of the carbon materials that are used as cathodes or additives^2,5^. It has been reported that different carbon morphologies could affect the formation of lithium oxide species, which influence the performance of Li–O_2_ batteries^2,5^.

**Figure 1:**
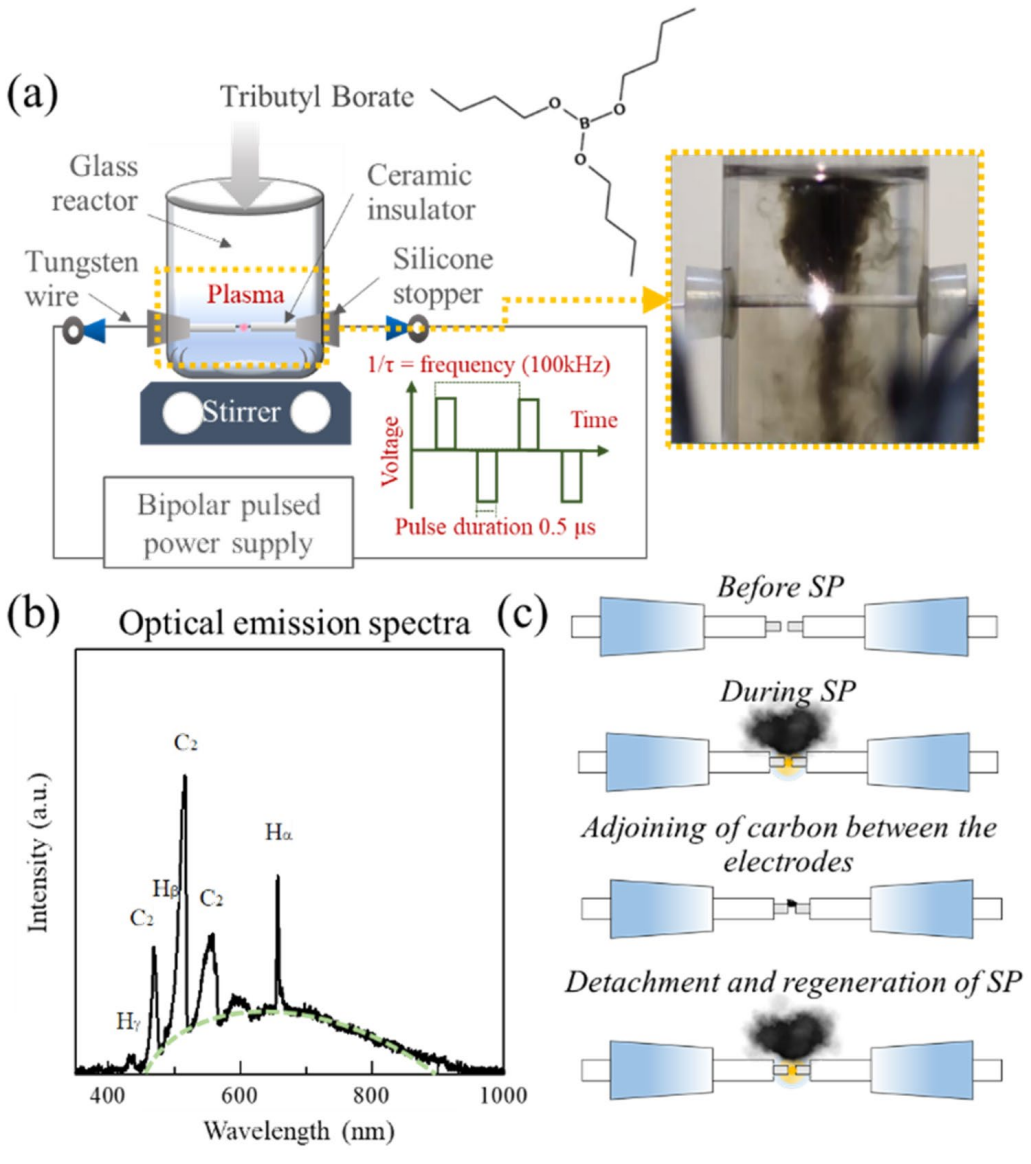
Illustration of (**a**) SP set up in this study with actual images of synthesis of carbon samples via SP, (**b**) OES measurement, and (**c**) possible formation of carbon samples.

Several techniques such as pyrolysis have been suggested for the preparation of carbon materials^6^. In-liquid plasma, the so-called solution plasma (SP), is a promising facile method proposed for the production of carbon nanoparticles and their derivatives^7^. In the SP process, a bipolar pulsed power supply is used, and the electricity flows through the electrodes to the tips submerged under a solution, as shown in Fig. 1a^8^. Then, the generated free electrons collide with the surrounding molecules, leading to the dissociation of molecules and production of highly reactive species, whose type depends on the type of solution. For example, the SP discharge of benzene molecules was found to dissociate and generate H, H^+^, and C_2_ radicals, which later rapidly recombine and form carbon nanoparticles^9^. According to previous studies, SP has been effectively used for the synthesis of undoped and heteroatom-doped carbon materials, which have the potential for being used as both cathodic catalysts and catalyst supporting materials^10^. In addition, SP has been proven to facilitate the doping and tuning of the carbon material structure by adjusting the SP operating conditions or changing precursors. For example, in 2013, Kang and co-workers reported that the diameters of carbon nanoparticles generated from the plasma discharge of benzene could be changed depending on the pulsed frequency^11^. Later, Hyun et al. found that the chemical structures of precursors played an important role in the formation of carbon nanosheets, leading to the improved quality of 2D nanostructures such as graphene, via SP^12^. However, reports relating to the synthesis and investigation of the different reconstructed structures of SP-induced carbon materials are still rare and challenging.

In this study, graphite-like carbon was incidentally produced during the synthesis of amorphous carbon (which contained boron and tungsten carbide) via SP. Therefore, it was of interest to evaluate and compare the SP-induced graphite-like carbon and the amorphous carbon. Tributyl borate (TBB) was used as the precursor. The synthesis was conducted in one step at room temperature and atmospheric pressure without the addition of other chemicals. The dissociation and formation of C_2_ radicals were observed using optical emission spectroscopy (OES). Field emission scanning electron microscopy (FE-SEM), transmission electron microscopy (TEM), x-ray diffraction (XRD), Raman spectroscopy, and x-ray photoelectron spectroscopy (XPS) were conducted to confirm the morphological, structural, and chemical properties of the obtained carbon samples. Finally, Li–O_2_ battery tests were also performed.

## Results

When TBB was subjected to SP, carbon material (black solid content) was continuously produced (Fig. 1a). To observe the SP-induced reaction, optical emission spectra (OES) measurements were conducted, as shown in Fig. 1b; the main spectra that were observed belonged to H and C_2_ radicals, which are important species for the synthesis of carbon materials via SP. During SP, the carbon content occasionally adjoined between electrodes, which led to the disappearance of plasma, before it was detached (Fig. 1c). After detachment, the plasma regenerated, and the carbon content continued to generate. After the SP process, two different types of carbon samples, carbon sample dispersed in solution and carbon that precipitated at the bottom of the SP reactor, were observed. The carbon sample dispersed in the solution was assigned as the in-liquid carbon (LC), while the carbon at the bottom of the SP reactor was assigned as precipitated carbon (PC). According to visual observation, LC was found to be carbon that was produced during plasma generation, while PC was found to be generated from the adjoining between the electrodes.

**Figure 2:**
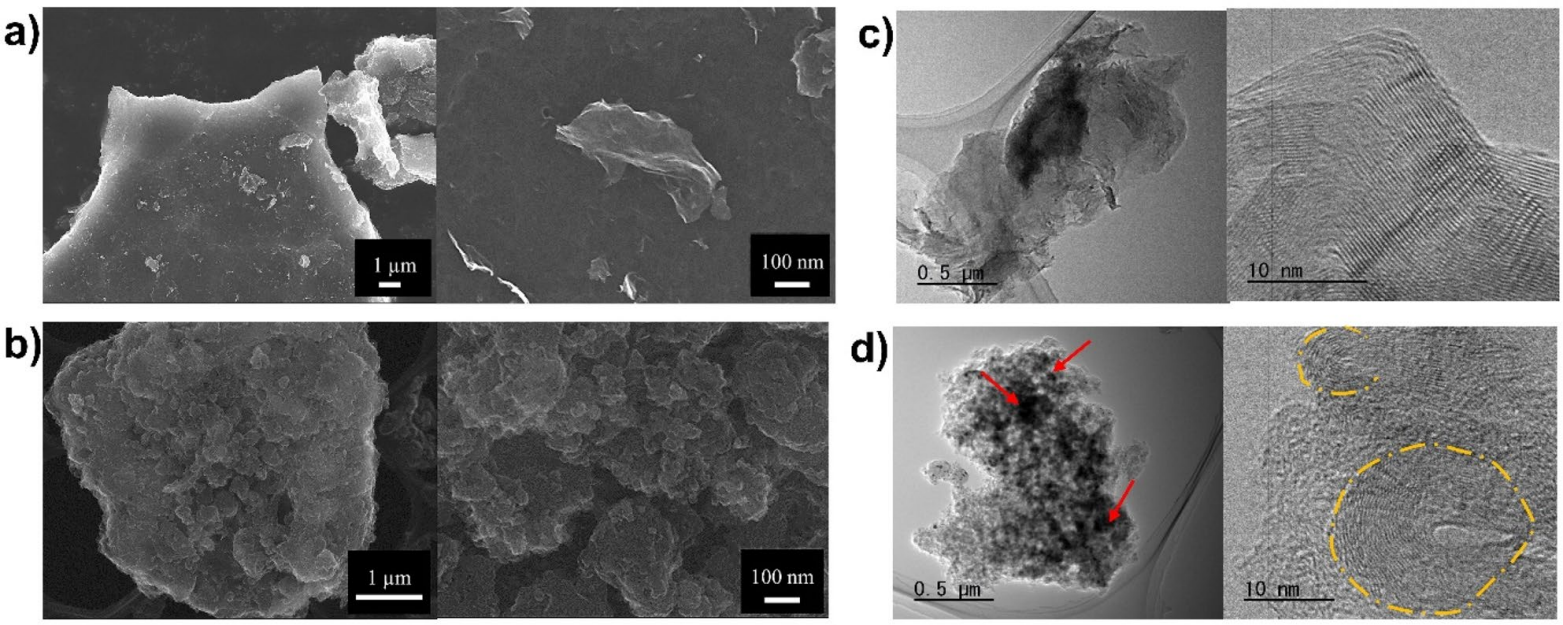
FE-SEM images of (**a**) PC at the magnitudes of × 5 k and × 100 k and (**b**) LC at the magnitudes of × 5 k and × 100 k, synthesized from TBB via SP at a frequency of 100 kHz for 60 min. TEM and high-resolution TEM images of (**c**) PC and (**d**) LC synthesized from TBB via SP at a frequency of 100 kHz for 60 min.

Morphology of the obtained product, studied by observing the FE-SEM images of PC at magnifications of 5 k and 100 k (Fig. 2a), was both stacked and separated thin sheets of carbon. On the other hand, FE-SEM images of LC (Fig. 2b) show the agglomeration of carbon nanoparticles with spherical and irregular shapes. Furthermore, morphologies of PC and LC were investigated using TEM (Fig. 2c and d). According to the TEM results, PC displayed a flake-like structure, while LC exhibited aggregation of primary particles. In addition, small dark spots could be observed, especially in the TEM image of the LC (red arrow). These spots were believed to be tungsten-based particles, which might have been incidentally sputtered from the tungsten electrodes. In the high-resolution TEM images, LC was found to be composed of fullerene-like multilayer carbon (yellow dashed-line circle) surrounded by a disordered amorphous phase. In the high-resolution TEM of PC, the carbon sheets were found to have a stacked structure with approximately 45 graphitic layers with approximately 0.34 nm distance between the planes.

**Figure 3:**
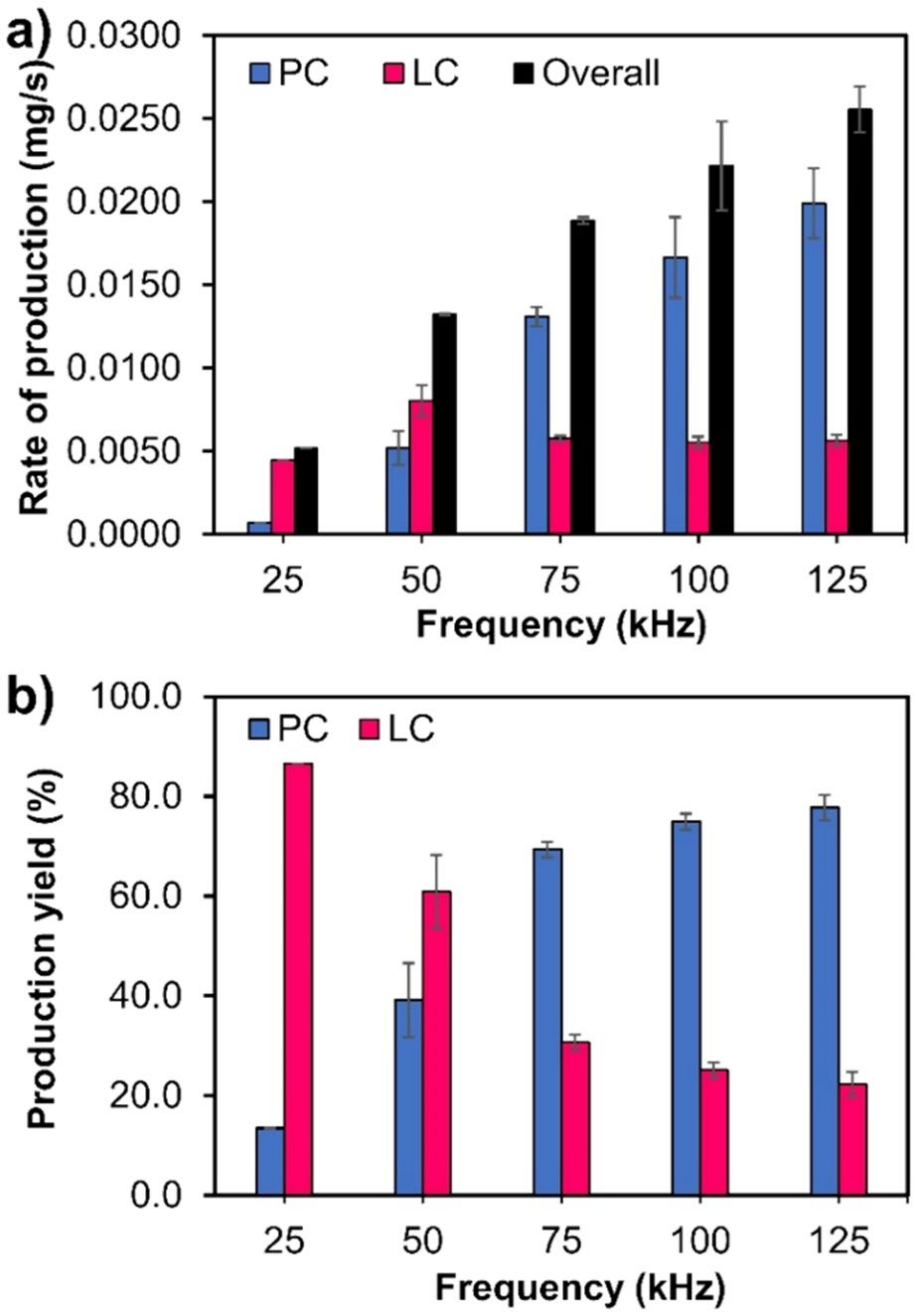
(**a**) Rate of production and (**b**) production yield of PC and LC synthesized from TBB via SP at frequencies of 50, 75, 100, and 125 kHz for 60 min.

Triethyl borate (TEB) and trimethyl borate (TMB), which have shorter hydrocarbon chains than TBB, were also used as precursors in this study. The amounts of both PC and LC obtained from SP using TEB at 100 kHz for 60 min were relatively low. By using TBB, approximately 60 mg of PC and 20 mg of LC were produced, while by using TEB, only 10 mg of PC and a negligible amount of LC were produced, under the corresponding conditions. When TMB was used during SP, carbon materials were not produced; therefore, mainly TBB was studied. When TBB was treated with SP for 60 min at frequencies of 50, 75, 100, and 125 kHz, the results showed that the production rate of PC increased with increasing SP operating frequency, while the production rate of LC remained almost constant (Fig. 3a). In addition, with increasing SP operating frequency, the yield of PC increased, while that of LC decreased. (Fig. 3b).

**Figure 4:**
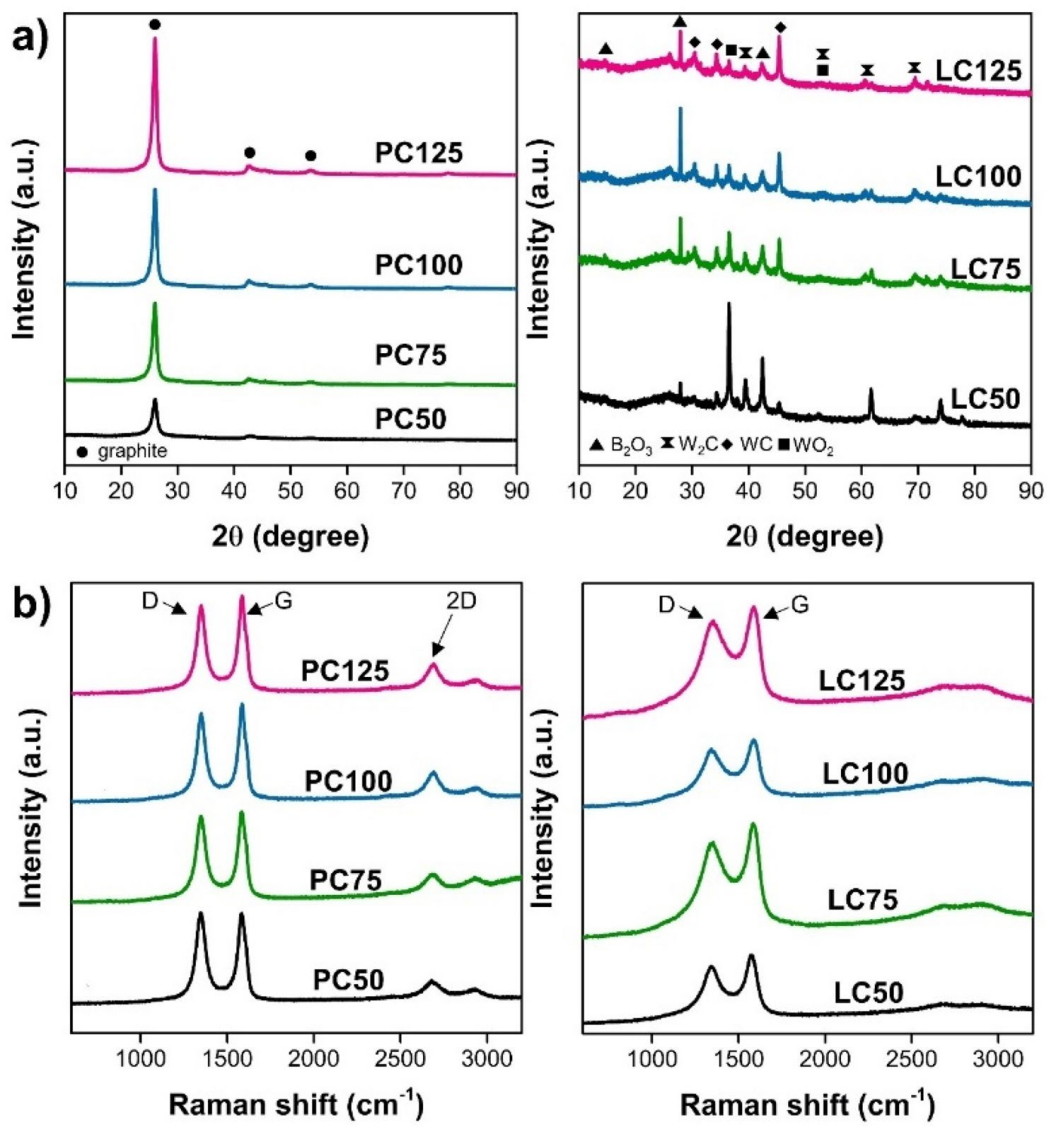
(**a**) XRD patterns and (**b**) Raman spectra of PC and LC synthesized from TBB via SP at frequencies of 50, 75, 100, and 125 kHz for 60 min. (*Note* PC50, PC75, PC100, and PC125 refer to the PC samples prepared via SP at frequencies of 50, 75, 100, and 125 kHz, respectively. LC50, LC75, LC100, and LC125 refer to the LC samples prepared via SP at frequencies of 50, 75, 100, and 125 kHz, respectively).

For structural information, the XRD spectra of both LC and PC synthesized from TBB via SP for 60 min were obtained (Fig. 4a). PC exhibited a very sharp diffraction peak at approximately 2θ = 26° and two additional peaks at 44° and 54°, corresponding to the 002, 101, and 004 reflections of graphite, respectively. Meanwhile, LC exhibited a broad diffraction peak at approximately 2θ = 26°, which is characteristic of an amorphous phase. However, LC still showed several additional peaks at 15.1°, 27.9°, and 40°. Raman spectra of both LC and PC synthesized from TBB via SP for 60 min were also obtained (Fig. 4b). The Raman spectra of these two samples exhibited various main feature bands, including the D-band (~ 1340 cm^–1^), A-band (~ 1480 cm^–1^), G-band (~ 1578 cm^–1^), and 2D-band (~ 2660 cm^–1^). In addition, the relative intensity ratio of the D-band to the G-band (I_D_/I_G_) is widely known to be an indicator for quantitatively measuring the structural defects in carbon materials. The I_D_/I_G_ of LC was approximately 0.85, while the I_D_/I_G_ of PC was slightly larger than 0.90 (Table 1).

**Table 1:** Intensity ratio of D and G bands (I_D_/I_G_) from Raman spectroscopy, and surface element compositions and doping concentration from XPS measurement of LC and PC synthesized from TBB via SP at frequencies of 50, 75, 100 and 125 kHz for 60 min (Note: at% means atomic percent).

Samples	I_D_/I_G_	XPS				
		Composition (at%)			Ratio	
		C	O	B	O/C	B/C
LC50	0.85	75.54	19.84	4.62	0.263	0.061
LC75	0.85	73.76	26.11	0.13	0.354	0.001
LC100	0.86	77.25	19.83	2.92	0.257	0.037
LC125	0.89	69.36	24.82	5.82	0.358	0.084
PC50	1.00	90.44	9.23	0.33	0.102	0.003
PC75	0.95	83.79	15.98	0.23	0.191	0.003
PC100	0.90	77.46	22.42	0.12	0.289	0.002
PC125	0.90	74.83	25.13	0.04	0.336	0.001

**Figure 5:**
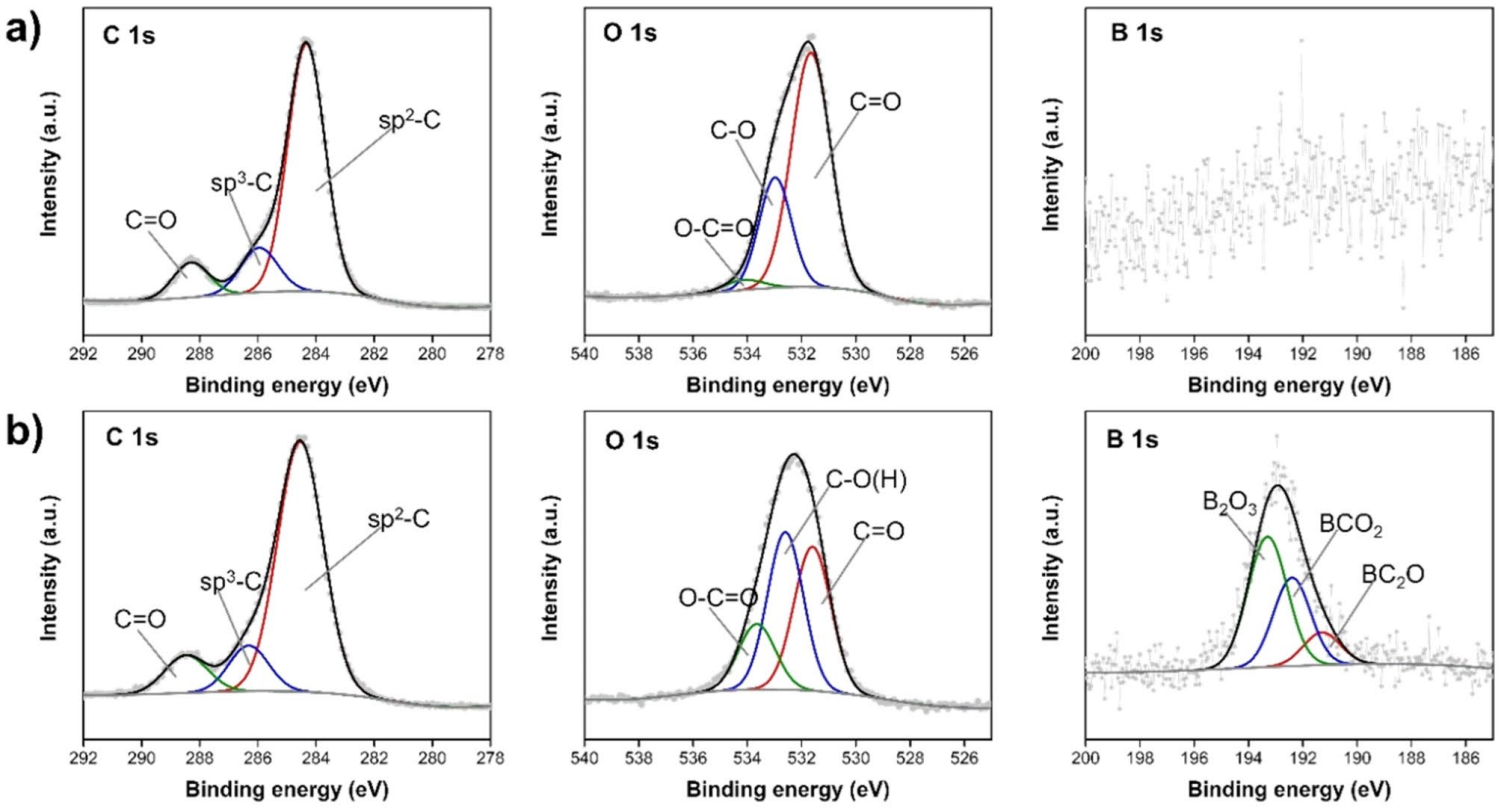
C 1 s, O1s, and B1s XPS spectra of (**a**) PC and (**b**) LC synthesized from TBB via SP at frequency of 100 kHz for 60 min.

Furthermore, the elemental compositions of the bulk and surface of LC and PC were evaluated through elemental analysis (EA) (Supplementary Table S1) and XPS spectroscopy (Table 1). The EA results showed that the bulk of LC was composed of carbon and hydrogen at 56 and 2 wt%, respectively, while most of the PC had a carbon content of 91 wt%. Because TBB contains boron, it was expected that the obtained carbon products could be boron-doped. XPS measurements were used to further confirm the boron content and bonding configuration on the surfaces of both LC and PC. The XPS results revealed that LC had a higher boron content than PC. Furthermore, according to the high-resolution XPS spectra (Fig. 5), the C 1 s peaks of both PC and LC consisted of different components that could be deconvoluted into *sp*^2^ (284.6 eV), *sp*^3^ (285.8 eV), and C = O (287.0 eV). For O 1 s, there were three main deconvoluted components, i.e., C = O (531.8 eV), C–O(H) (533.0 eV), and O–C = O (534.9 eV) for both PC and LC. For B 1 s, the deconvoluted components could be identified only for LC, because the B 1 s signal of PC was very low. The deconvoluted components of the B 1 s peak could be divided into three components, i.e., BC_2_O (191.1 eV), BCO_2_ (192.2 eV), and B_2_O_3_ (193.6 eV).

**Figure 6:**
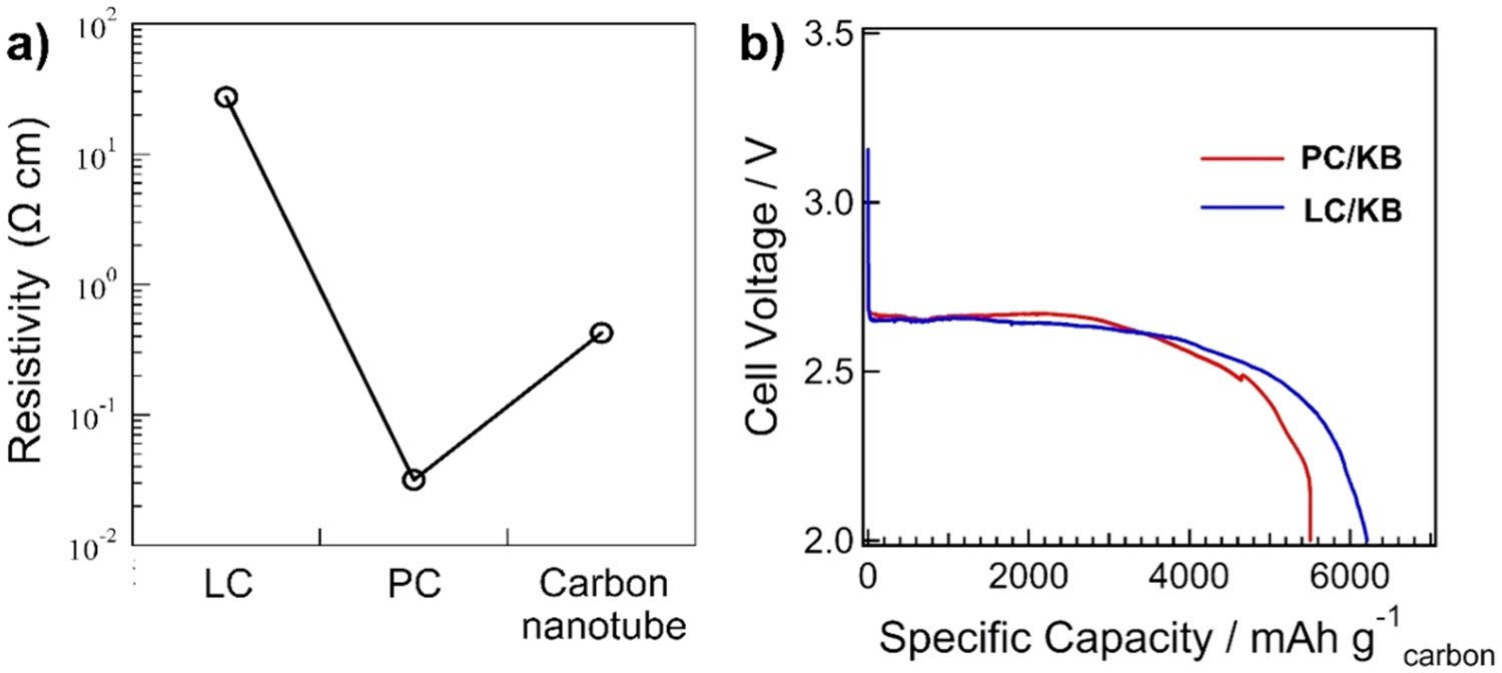
(**a**) Electronic conductivity and (**b**) initial full discharge curves of battery cells with PC/KB and LC/KB cathodes at current density 100 mA g^–1^ and cut-off voltage of 2 V.

**Figure 7:**
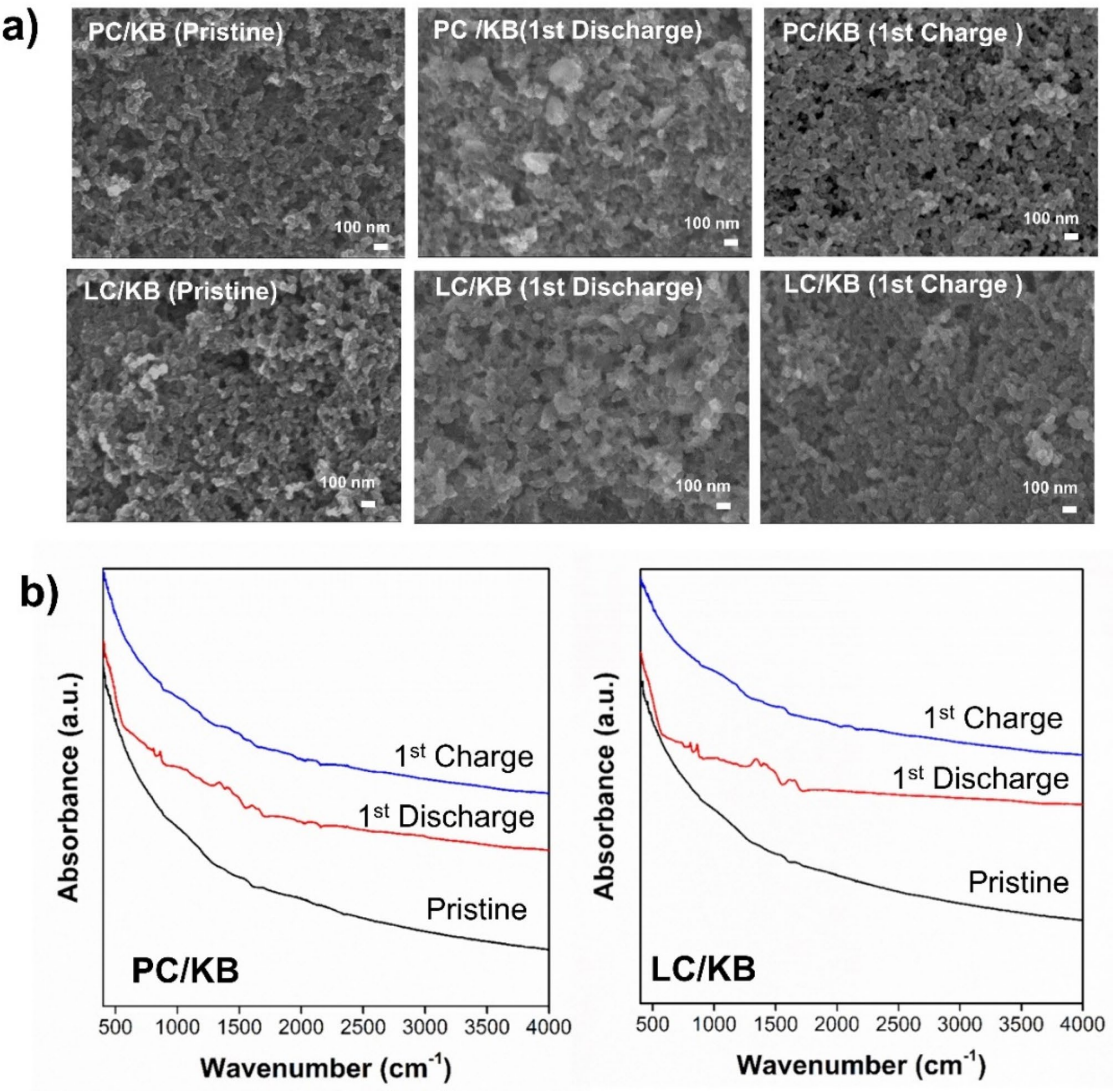
(**a**) FE-SEM images and (**b**) FT-IR spectra of PC/KB and LC/KB cathodes from the initial full discharged cells at current density of 200 mA g^–1^ and cut-off voltage of 2 V.

The electrical resistivities of LC and PC were also determined (Fig. 6a) in comparison with cup-stacked carbon nanotubes, which are believed to be carbon materials with high electrical conductivity. Among the tested samples, PC showed the lowest resistivity and highest electrical conductivity, while LC exhibited the highest electrical resistivity. Because PC and LC have significantly different characteristics, the evaluation of PC and LC as additive materials for cathodes in Li–O_2_ batteries could be interesting. PC had a graphite-like structure and notable electron conduction, while LC had a turbostratic structure with higher porosity and boron content than PC (Supplementary Figure S3). The full discharge measurement of the battery cells with the cathode prepared from the composites of KB and either PC or LC at a ratio of 1:1, assigned as PC/KB and LC/KB, respectively, was performed at a cut-off voltage of 2.0 V at a current density of 100 mA g^–1^ (Fig. 6b). The battery cell with the LC/KB cathode yielded an initial capacity of approximately 6,208 mAh g^–1^_,_ while PC/KB produced a lower initial capacity of 5,501 mAh g^–1^. The evaluation of the products generated on the cathodes was carried out after the initial discharge–charge with a cut-off capacity of 1000 mAh g^–1^ at a current density of 200 mA g^–1^ using FE-SEM and FT-IR. According to the FE-SEM images (Fig. 7a), toroid-like particles were observed on the surface of the PC/KB electrode after discharging, whereas they were rarely observed on the surface of the LC/KB cathode. According to the Fourier Transform Infrared Spectroscopy (FT-IR) result (Fig. 7b), the peaks appeared at approximately 550, 1500, and 1700 cm^‒1^ for the discharged electrodes and disappeared after charging.

## Discussion

During the SP treatment of TBB, the carbon materials could be produced from the plasma zone through the decomposition and recombination of hydrocarbon molecules^13^. The decomposition and recombination of hydrocarbon molecules could be confirmed by the presence of C_2_ during the reaction, which has been reported to play an important role in the formation of carbon nanoparticles^9^. According to the FE-SEM, TEM, and XRD results, the morphology of the PC was found to be similar to that of the graphene-like carbon sheets reported in previous works, while LC mainly contained turbostratic carbon surrounding multi-layer fullerene-like carbon, which is generally produced by SP^1,14–19^. In addition, the Raman results suggested that PC contained defective crystal phases, but LC mainly contained an amorphous phase embedded with small, ordered carbon structures. The defects in the crystal planes of PC could have been formed via the oxidation reaction owing to the presence of oxygen atoms in the system, which could be excited by the SP into becoming oxygen active species and reacting with the carbon structure^13^. This result was consistent with the XPS results which indicated that PC had a carbon framework with larger defects containing C=O groups in the plane and at the edges. In addition, the XRD results also showed that the LC contained tungsten-based nanoparticles, such as WO_2_, WC, and W_2_C^20–26^. These nanoparticles can result from the erosion of electrodes and formation of by-products, such as tungsten carbide, depending on the SP operating conditions^17^.

Moreover, various boron-containing precursors, such as TEB and TMB, were used for the synthesis of carbon nanoparticles for comparison with TBB. The results showed that the decomposition of TEB and TMB, which have shorter chain lengths than TBB, make C_2_ more difficult to obtain because there are fewer C–C bonds in their chemical structures. Similar to the degradation of a polymer, molecules with shorter chains might have high mobility, leading to short-time contact with plasma and lower probability of being attacked by the highly reactive species^27^. Thus, the structure, such as the linear chain length of hydrocarbon, of aliphatic precursors could be suggested as a very important factor in the formation of carbon nanoparticles.

The influence of SP operating conditions was investigated for further study of carbon formation using TBB. The increase in SP operating frequency resulted in the formation of PC with higher crystallinity and lower defects, whereas it did not show significant effects on LC. From the XPS measurements (Table 1), SP was found to deposit boron species on LC but could not dope boron species in the carbon framework, because the signal of B–C was not present in the B 1 s peak. It was also found that the existing boron in the LC was B_2_O_3_. The presence of B_2_O_3_ in the LC might be due to the residues of TBB that were left after the decomposition of the outer hydrocarbon chains to form the PC. In addition, the presence of B_2_O_3_ in the LC could also be due to side reactions and oxidation because SP is usually conducted in the open reactor surrounding the atmospheric environment where O_2_ is present. O_2_ can get absorbed into the reaction medium and participate in the reaction. However, for the PC, the B 1 s peak was hardly observed, and as the SP operating frequency increased, the boron content decreased. This suggests that the direct contact of carbon contents with the electrical discharge at the tips of electrodes could not effectively result in the doping of boron. Based on the evidence obtained in this work, the formation of these two carbon materials by the SP might occur via two different pathways according to the characteristic feature of SP, in which the plasma is formed by a discharge and sustained in the bubbles^28–30^. The LC might be generated at interfaces between bubbles and the surrounding solution from the excitation and combination of carbon molecules via the collisions of highly reactive species, induced by the electrical discharge, and carbon molecules. On the other hand, during the SP, the temperature of plasma at the tip of the electrodes reached up to ∼ 4000 K or higher, while that of the surrounding solution remained at room temperature^31^. Therefore, it is possible that when the SP-induced carbon nanoparticles are attached to the electrodes, the high temperature might cause melting and fusing of particle layers, resulting in the rearrangement and formation of the carbon with high crystallinity^32^. This carbon with high crystallinity is referred to as PC. This phenomenon is similar to the graphitization of amorphous carbon upon heating to high temperatures and applying an electric current^32^. Moreover, it is also possible that PC is formed from the decomposition of TBB and recombination at the plasma zone. It has been reported that linear hydrocarbon molecules could be decomposed into shorter molecules such as C_2_ before recombination in the plasma zone where the plasma temperature is high, similar to pyrolysis^9^. The higher plasma temperature between the gap of electrodes could lead to the generation of carbon materials with higher crystallinity, similar to carbon materials obtained from pyrolysis at very high temperatures^33,34^. In addition, due to the high temperature (∼ 4000 K) in the plasma zone, tungsten could be melted into metal atoms, and the atoms could diffuse to the liquid interface before clustering to form metal nanoparticles. As a result, tungsten-based nanoparticles were rarely observed in the PC structure. However, even though the temperature of the plasma zone of SP was very high, the heat can be transferred from the plasma zone and reduced through the surrounding liquid^7^. Consequently, this can be considered as a benefit of SP because it can be used to synthesize carbon with high crystallinity and maintain the system at room temperature, unlike pyrolysis.

Moreover, a higher production yield of PC was observed with increasing SP operating frequency. This might be because the bipolar pulse generator was used in the SP system^11^. Therefore, the increasing frequency could increase the number of cycles of plasma discharge in the same duration and increase the plasma temperature due to increasing energy input (Supplementary Data Figure S3). The same result was also reported in a previous study in which the plasma discharge in the SP process with higher energy input could lead to the generation of more continuous short-range graphene layers, compared to that from the plasma discharge with lower energy^11^. This suggests that the production of high-crystallinity carbon, as well as the carbon structure, can be altered by adjusting the SP operating conditions. Moreover, the results of this study combined with other previous work could conclude that the chemical structure of the precursors plays a significant role in the structure of SP-generated carbon materials. Examples are shown in the Supplementary Data Table S2. Graphite-like carbon could be generated from aliphatic molecules, while both amorphous and sheet-like carbons could be generated from the aromatic molecules, depending on the number of carbons in the ring and double bonds.

Because of the different structures and properties of the obtained products, PC with a graphite-like structure and high electron conductivity and LC with a turbostratic structure with higher porosity and boron and WC content, the evaluation of PC and LC as the additive materials for the cathode in the Li–O_2_ battery was of interest. It has been reported that both the active sites and electronic conductivity of the carbon matrix play important roles in additive or cathodic materials. Qiao and co-workers showed that a low conductivity limited access to active sites^35,36^. The cathode should provide sufficient electrons, Li^+^, and O_2_ transport to form the desired product, i.e., Li_2_O_2_, and achieve an ideal cathode reaction (2Li^+^  + 2e^–^ + O_2_ ↔ Li_2_O_2_)^37^. Therefore, the obtained carbons were further evaluated as active material for the cathode in the Li–O_2_ battery. In the Li–O_2_ cells, the cathode prepared from the composite of KB and LC showed better performance than that of the KB and PC composite, which might be due to the presence of WC and higher porosity. WC has been reported as a cathode coating material that can lead to performance improvement in Li–O_2_ batteries^38^. Meanwhile, the formation of the desired product, Li_2_O_2_, which generally contains toroid-like particles^39^, was found to favor the cathode prepared from the composite of KB and PC compared to that prepared from the composite of KB and LC. It was because the electronic conductivity of PC was higher than that of the LC. The higher electronic conductivity should facilitate electron transfer, leading to the promotion of electrochemical reactions (2Li^+^  + 2e^–^ + O_2_ ↔ Li_2_O_2_) inside the Li–O_2_ cells. The FT-IR results also confirmed the formation of Li_2_O_2_, Li_2_CO_3_, and lithium acetate products after discharge^40,41^. The FT-IR spectrum of the discharged electrode (Fig. 7b) showed peaks at approximately 550, 1500, and 1700 cm^‒1^, indicating the formation of Li_2_O_2_, Li_2_CO_3_, and lithium acetate products, respectively. Upon charging, the FT-IR signal attributed to Li_2_O_2_ significantly decreased, indicating the disappearance of Li_2_O_2_, while the FT-IR signals of the Li_2_CO_3_ and lithium acetate products were still observable. They could be generated by the transformation of Li_2_O_2_ and LiO_2_, which were stabilized by the carbon surface at defects or functional groups, via an electrochemical reaction with the electrolyte^42,43^. Consequently, the graphite-like structure of carbon with high electronic conductivity could be a key factor for facilitating the formation of the desired Li_2_O_2_ in Li–O_2_ batteries, which might have a positive influence on the cycling performance (Supplementary Data Figure S5). From this work, it could be proposed that the combination of PC and LC could result in a satisfactory performance of the Li–O_2_ battery. Therefore, further studies to obtain graphite-like structured carbon with high porosity and high concentration of heteroatoms and WC should be conducted to achieve good electrocatalytic activity and cycling performance.

## Conclusion

Graphite-like and turbostratic carbon materials were simultaneously and successfully synthesized using the solution plasma (SP) from tributyl borate (TBB) within 60 min. There are two different pathways for the formation of carbon materials via SP that were proposed in this work: (1) the pyrolysis-like pathway at the plasma zone with high temperature, leading to the graphite-like undoped carbon, and (2) the polymerization pathway, resulting in turbostratic carbon containing boron and WC. The different reconstructed structures of these carbon materials were found to produce different physical and electrical properties, which required further tests with Li–O_2_ batteries. The graphite-like structure of carbon positively affected the formation of lithium oxide species and cycling performance, while the turbostratic-structured carbon containing heteroatoms and WC showed a relatively high discharge capacity. The results obtained in this work could serve as a guideline for the further development of cathodic materials used in Li–O_2_ battery applications. In addition, the SP could be considered as a promising tool for the production and modification of carbon materials not only for Li–O_2_ batteries but also for various other applications, which can meet economic feasibility and sustainability requirements.

## Methods

### Solution plasma (SP)

TBB (80 mL) was poured into the SP reactor, which was adapted from a 100 mL beaker and connected to a bipolar pulsed power supply (Kurita Seisakusho Co. Ltd.), as shown in Fig. 1. The stable plasma could be induced by providing a voltage of 1.5 kV to a pair of tungsten wire electrodes (diameter 1 mm, purity 99.9%, Nilaco Corp.). The pulse width was fixed at 0.5 μs, while the frequency was varied, i.e., 50, 75, 100, and 125 kHz. The reaction was allowed to proceed for 60 min. After the reaction, two types of carbon samples were observed, a carbon sample dispersed in the liquid phase and a carbon precipitate at the bottom of the reactor. The two samples were separated by pouring the solution into another container, which left only the carbon precipitate in the SP reactor. The dispersed carbon sample was collected by suction filtration and carefully purified using pure water, ethanol, and acetone. Both samples were then dried in an oven at 100 °C for 12 h and retained for further characterization.

### Li–O_2_ battery test

The obtained carbon samples were mixed with Ketjenblack (KB) to make the carbon composites at a mass ratio of 1:1. Then, the carbon composite was dissolved in a mixture of polyvinylidene difluoride (PVDF) and *N*-methylpyrrolidone (NMP) to form a carbon slurry. The mass ratio of the carbon composite to PVDF was 9:1. The carbon slurry was sonicated for 30 min to obtain a homogeneous carbon slurry before being applied to TGP-H-060 to prepare the cathode. The obtained cathode was dried in a vacuum oven at 120 °C overnight to remove any residual solvent. Tetraethylene glycol dimethyl ether (TEGDME) was dried for several days over freshly activated molecular sieves (type 4 Å) before use. An ether-based electrolyte was prepared in an Ar-filled glovebox (H_2_O and O_2_ levels < 1 ppm) by dissolving 1 M lithium bis(trifluoromethanesulfonyl)imide in TEGDME. Swagelok-type cells were assembled in an Ar-filled glovebox (H_2_O and O_2_ levels < 1 ppm) using lithium metal foil as the anode, a glass-fiber membrane immersed in the electrolyte as the separator, and Ni foam as the gas diffusion layer. Discharge and charge profiles were collected under oxygen gas flow using a battery discharge–charge system (HJ1005SD8 and HJ1001SD8C, Hokuto Denko). For the evaluation of cycling performance, the coulombic efficiency (CE) was also calculated by the ratio of charge capacity at 4.5 V and discharge capacity^11^. For cathode characterization, the cells were first transferred to the Ar-filled glove box and disassembled inside it to extract the cathodes. The cathodes were then rinsed with DME and dried for further characterization. FE-SEM was employed to observe the cathode and discharge products. FT-IR (IRTracer-100, Shimadzu) with an attenuated total reflectance (ATR) accessory, where the samples were mounted on a diamond crystal, was also employed.

## Supplementary Information

Supplementary Information
