# Supplementary material for: Simultaneous synthesis of graphite-like and amorphous carbon materials via solution plasma and their evaluation as additive materials for cathode in Li–O2 battery
Source: Sci Rep. 2021 Mar 18;11:6261. doi: 10.1038/s41598-021-85392-2 (PMC7973806; doi:10.1038/s41598-021-85392-2)
Supplement: Supplementary file 1 — Supplementary Information [file 41598_2021_85392_MOESM1_ESM.docx]

Supplementary data

**Simultaneous synthesis of** **graphite-like and amorphous carbon materials via solution plasma and their evaluation as additive materials for cathode in Li-O_2_ battery**

**Materials and chemicals**

Tributyl borate (C_12_H_27_BO_3_, purity >98.0%), triethyl borate (C_6_H_15_BO_3_, purity >97.0%), and trimethyl borate (C_3_H_9_BO_3_, purity >98.0%) were purchased from Tokyo Chemical Industry Co., Ltd. Ethanol (C_2_H_5_OH, purity 99.5%) and 0.1 M potassium hydroxide (KOH) aqueous solution was purchased from Kanto Chemical. Nafion solution (5 wt% in a mixture of lower aliphatic alcohols and water) was purchased from Sigma-Aldrich. All reagents were used without further purification. Ultrapure water (18.2 MΩ cm) was produced from an RFD250NB Aquarius water purification system. For a primary battery test, tetraethylene glycol dimethyl ether (C_10_H_22_O_5_, 99%) and polyvinylidene fluoride were supplied by Sigma-Aldrich. Ni foam (16 mm in diameter) from Nilaco, Ketjenblack, KB (EC600JD), glass fiber separator (GF/A, 21mm in diameter) from Whatman, and lithium metal foil anode (15mm in diameter) from Honjo Metal and carbon paper (TGP-H-060, 16mm in diameter) from Toray were used. N-metyl pyrrolydone (C_5_H_9_NO) and lithium bis(trifluoromethane sulfonyl)imide (LiC_2_F_6_NO_4_S_2_, 99.7%) were purchased from Wako Pure Chemical Industries and Kanto Chemical, respectively. The cup-stacked carbon nanotubes were purchased from GSI Creos. According to the maker specification, the outer and inner diameter was 80-100 nm and 50-70 nm, respectively. Its length was 5 µm.

**Characterizations**

Morphologies and element mapping of the obtained samples were observed by field emission scanning electron microscopy (FE-SEM; JEOL JSM-7610F microscope). Moreover, transmission electron microscopy (TEM) and high-resolution TEM images of the obtained samples were also obtained by using a TEM microscope (JEOL JEM-2100 microscope) at an accelerating voltage of 200 kV. The phase structures were analyzed by using an X-ray diffractometer (XRD; Rigaku SmartLab X-ray diffractometer) with monochromatic Cu Kα radiation (λ = 0.154 nm) operating at 40 kV and 40 mA (1.6 kW). Raman spectrometer (JASCO NRS-5100 spectrometer) was used to record the Raman spectra of the obtained carbon samples with a laser-excitation wavelength of 532.11 nm. X-ray photoelectron spectroscopy (XPS; JEOL JPS-9010MC spectrometer) was used to study the chemical compositions and bonding states of the obtained samples. The XPS spectrometer with monochromatic Mg Kα radiation (1253.6 eV) as an excitation source under ultra-high vacuum conditions. The operating emission current and anode voltages were 25 mA and 10 kV, respectively. Furthermore, the time-averaged optical emission spectrum (OES) of the discharge was observed with an optical spectrometer (HR2000+CG-UV-NIR; Ocean Optics). The specific surface area was investigated by employing the nitrogen absorption-desorption method (BET, Brunauer Emmett Teller) performed on a TriStar-Ⅱ3020 analyzer equipped with VacPrep 061LB at 77 K. The carbon samples were firstly degassed at 100 °C for 12 h under a vacuum before the measurements. The specific surface area was determined using the Brunauer–Emmett–Teller (BET) method in the relative pressure (*P*/*P*_0_) range of 0.05–0.30. The total pore volume and pore size distribution were evaluated by the Barrett–Joyner–Halenda (BJH) method. The production rate and percentage of production yield were calculated by the following equations:

$\text{Production rate = }\frac{\text{W}_{\text{i}}}{t}$ (1)

$\text{Percentage of production yield = 100 ×} \frac{\text{W}_{\text{i}}}{\text{W}_{\text{t}}}$ (2)

where *W_t_* is the total weight of the obtained carbon sample in the system, *W_i_* is the weight of either the carbon sample dispersing in the liquid phase or the precipitated carbon sample at the bottom of the reactor, while *t* is the reaction time. Electrical conductivity was measured in a purpose-built pressure cell. The cell consists of polytetrafluoroethylene (PTFE) die, and a stationary and moveable piston made of copper. Both stationary and moveable pistons have a diameter of 4.93 mm, while the cylindrical hole of the die is 5.00 mm in diameter. The powder of carbon sample was introduced into the die which was installed on the stationary piston and then covered with the movable piston to facilitate the fixation of copper wires used for the electric measurement. The pressure on the device was applied using a Riken Seiki mini-press set equipped with a type P-16B hydraulic hand pump 50 MPa. The sample was compressed with thresholds of 0.7 MPa and measured for the thickness of powder. The currents were observed from a regulated DC power supply (DCP3005, As one, Japan) at four different applied voltages. The obtained currents were plotted against applied voltages, to obtain resistance. The conductivity (*σ*) was calculated based on a reciprocal of resistance, according to *σ* = l/(R · A), where l is the powder column height obtained from the piston displacement, R is the resistance and A refers to the cross-sectional area of the piston.

Table S1: Bulk element compositions from CHN analysis of LC and PC synthesized by SP at the frequency of 100 kHz for 60 min

| Samples | EA (wt%) | | | | |
| --- | --- | --- | --- | --- | --- |
|  | C | | H | |  |
| LC | 56.5 | | 2.2 | |  |
| PC | 91.1 | | 0.1 | |  |
|  |  |  | |  | |

**
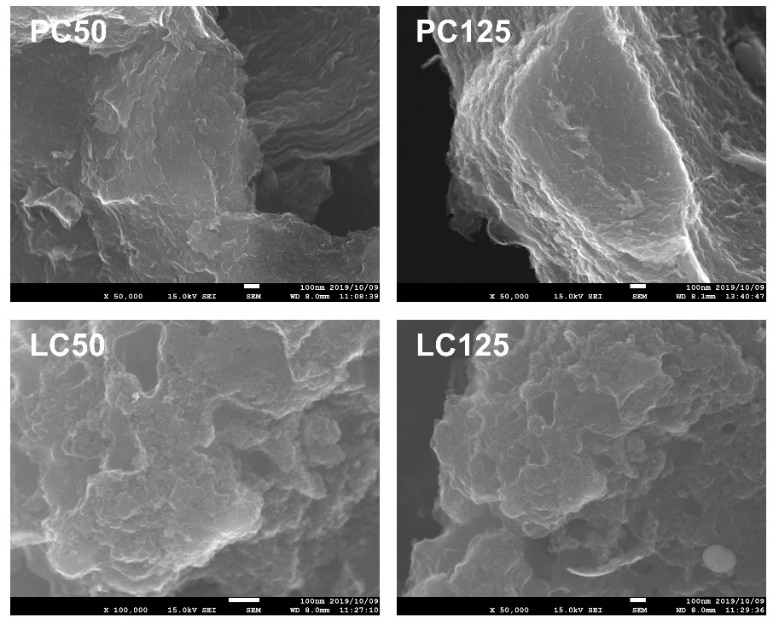
**

Figure S1: FE-SEM images of PC and LC at the magnitude of ×50k, synthesized from TBB via SP at the frequency of 50 kHz and 125kHz for 60 min.

**
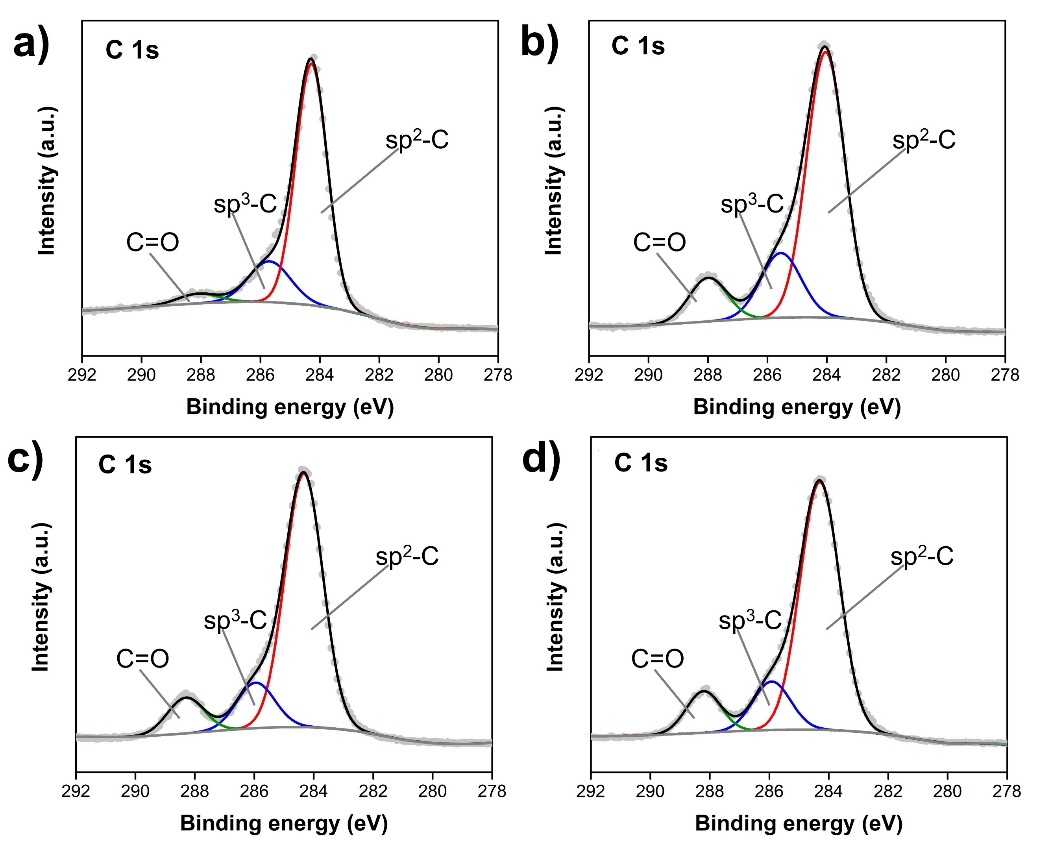
**

Figure S2: C 1s XPS spectra of PC and LC, synthesized from TBB via SP at the frequency of (a) 50 kHz, (b) 75 kHa, (c) 100kHz and (d) 125 kHz for 60 min.

To investigate the textural properties of the obtained carbon samples, the N_2_ adsorption analysis using the BET method was used to determine the specific surface area (*S*_BET_). Figure S3(a) and (b) exhibits the N_2_ adsorption and desorption isotherms and pore size distribution of PC and LC, respectively. The *S*_BET_ values of PC were found to be 9.3 m^2^ g^–1^, while that of LC equaled 41.1 m^2^ g^–1^. LC possessed more pores and a wide range of pore size distribution especially pores with sizes ranging from 10 nm to 60 nm. Meanwhile, the pores that appeared in PC exhibited a relatively narrow pore size distribution with a size of approximately 10 nm. In the previous study, it was found that the broader pores made the deeper diffusion and permeation of electrolyte and also had an advantage over the release of the generated gas inside the cathodic carbon materials.^1^ Consequently, in term of surface area and porosity, LC should show high potential to be used as a cathode for Li-O_2_ cell, compared to PC.

**
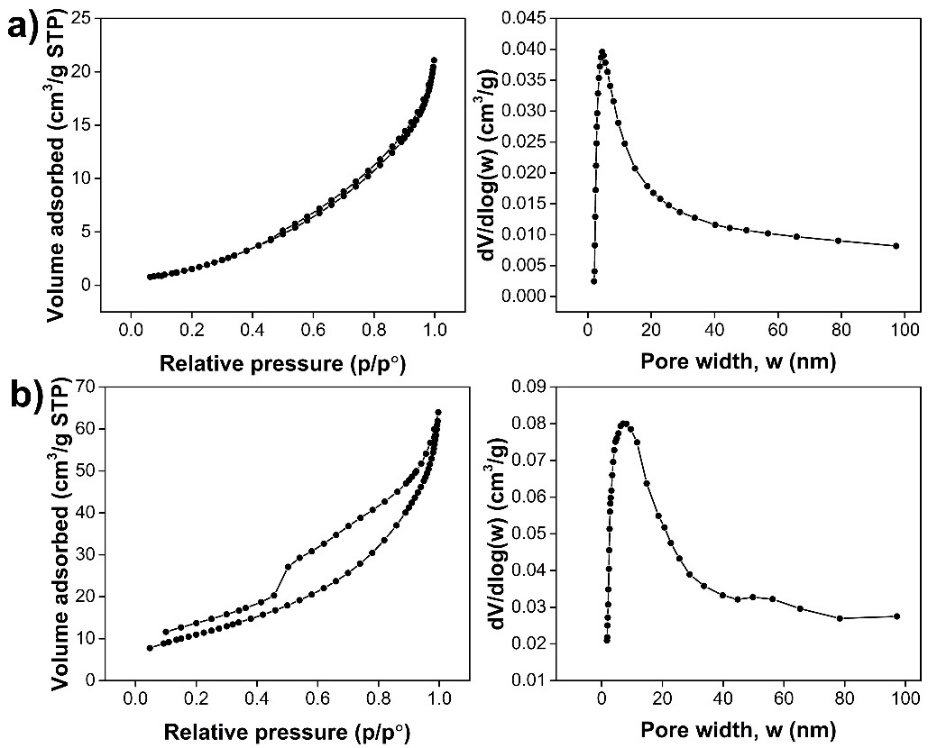
**

Figure S3: N_2_ adsorption-desorption isotherm and pore size distribution of (a) PC and (b) LC, synthesized from TBB via SP at the frequency of 100kHz for 60 min.

Figure S4: The first charge-discharge voltage plateau of pristine KB, PC/KB, and LC/KB at a cut-off capacity of 1000 mAh g^–1^ and a current density of 200 mA g^–1^.

The discharge voltage plateau of pristine KB was ~2.8 V, slightly higher than that of PC/KB and LC/KB. The charge voltage plateau of pristine KB was ~4.0 V, slightly lower than that of PC/KB and LC/KB. This could refer that the performance of PC/KB and LC/KB was slightly lower than that of pristine KB. In general, the suitable cathode materials for Li-O_2_ battery should have high porosity. According to the N_2_ adsorption-desorption isotherm result, PC and LC still has relatively low porosity. However, according to the previous work, the discharged cathodes using pristine KB were found to have grit-like agglomerates on the surface,^2^ which is less favorable, compared to the formation of the toroid-like Li_2_O_2_ crystals during discharging. When the PC was added to KB, it was surprisingly found that the toroid-like Li_2_O_2_ crystals were obtained during discharging.


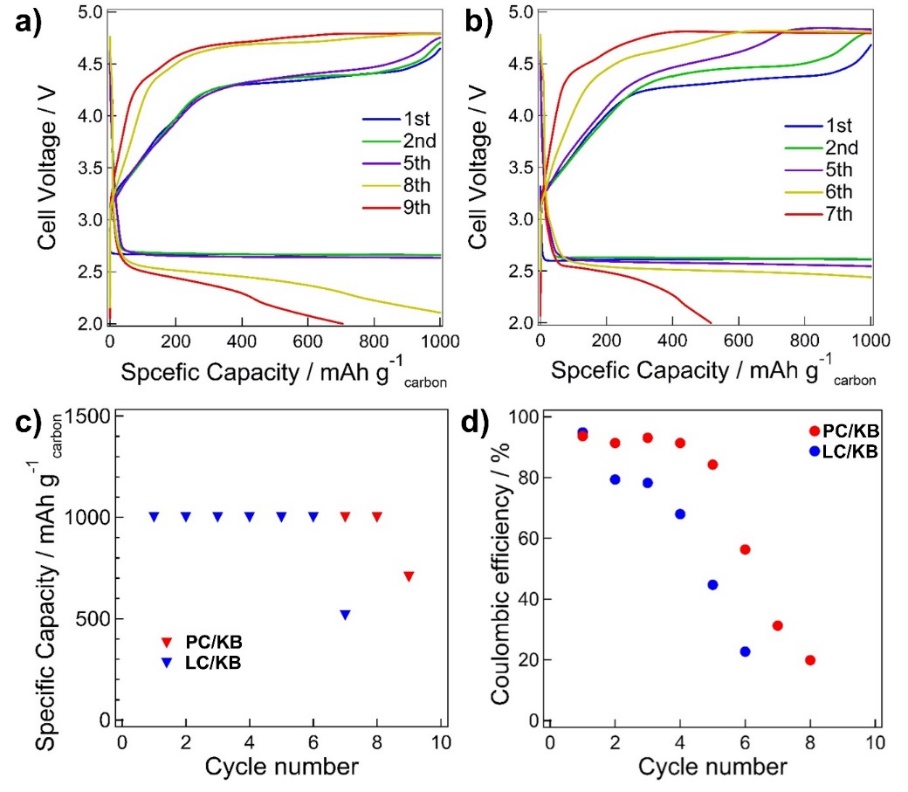


Figure S5: Cycling performances of Li-O_2_ with (a) PC/KB and (b) LC/KB electrodes, and their (c) specific capability and (d) Coulombic efficiency at a cut-off capacity of 1000 mAh g^–1^ and a current density of 200 mA g^–1^.

As the result in Fig. S5, the cycling performances of Li-O_2_ with PC/KB and LC/KB electrodes are shown. Li-O_2_ with PC/KB revealed to be higher cycling performance than that with LC/KB. However, the cycling performance of Li-O_2_ with both PC/KB and LC/KB was relatively low, compared to some previous works.^3-4^ According to the previous work, it was suggested that carbon materials that have few functional groups and defects showed more excellent stability, compared to carbon materials with high defects.^4^ It was believed that the high density of defects on the carbon electrode surface could hinder superoxide-toperoxide conversion via disproportionation.^5^ LiO_2_ is preferably trapped by the defects on the surface of the carbon and then transform to Li_2_CO_3_, leading to the decrease cycling performance of Li-O_2_. Therefore, the obtained PC is still required to be developed to reduce the defects and modified with a proper functional group, aiming to the higher cycling performance.

Table S2: Examples of SP-induced carbon materials from different precursors in the previous studies

| Precursors | SP conditions | Products | Ref. |
| --- | --- | --- | --- |
| Benzene | 25 and 65 kHz, 2 μs, 20 min, W electrodes | Amorphous carbon nanoparticles (25 kHz), turbostratic carbon nanoparticles (65 kHz) | ^6^ |
| Toluene | 100 kHz, 0.5 μs, 60 min, W electrodes | Turbostratic carbon nanoparticles | ^7^ |
| 2-pyrrolidone | 200 kHz, 1 μs, 5 min, W electrodes | Highly ordered nitrogen-carbon nanosheets | ^8^ |
| Pyrrole | 200 kHz, 1 μs, 5 min, W electrodes | Amorphous nitrogen-carbon nanoparticles | ^8^ |
| *N,N*-dimethylformamide | 100‒200 kHz, 1 μs, 60 min, Cu electrodes | N-doped few-layer graphene on Cu nanoparticles | ^9^ |
| Trimethyl borate | 100 kHz, 0.5 μs, 60 min, W electrodes | N/A | This work |
| Triethyl borate | 100 kHz, 0.5 μs, 60 min, W electrodes | Only graphite-like carbon, very small amount | This work |
| Tributyl borate | 100 kHz, 0.5 μs, 60 min, W electrodes | Graphite-like and amorphous carbon | This work |

**References**

1. Wang, J.; Wei, Z.; Wang, H.; Chen, Y.; Wang, Y., CoOx–carbon nanotubes hybrids integrated on carbon cloth as a new generation of 3D porous hydrogen evolution promoters. *Journal of Materials Chemistry A* **2017,** *5* (21), 10510-10516.
2. Lin, H.; Liu, Z.; Mao, Y.; Liu, X.; Fang, Y.; Liu, Y.; Wang, D.; Xie, J., Effect of nitrogen-doped carbon/Ketjenblack composite on the morphology of Li2O2 for high-energy-density Li–air batteries. *Carbon* **2016,** *96*, 965-971.
3. Kim, B. G.; Jo, C.; Shin, J.; Mun, Y.; Lee, J.; Choi, J. W., Ordered Mesoporous Titanium Nitride as a Promising Carbon-Free Cathode for Aprotic Lithium-Oxygen Batteries. *ACS Nano* **2017,** *11* (2), 1736-1746.
4. Liu, Y.; Wang, L.; Cao, L.; Shang, C.; Wang, Z.; Wang, H.; He, L.; Yang, J.; Cheng, H.; Li, J.; Lu, Z., Understanding and suppressing side reactions in Li–air batteries. *Materials Chemistry Frontiers* **2017,** *1* (12), 2495-2510.
5. Belova, A. I.; Kwabi, D. G.; Yashina, L. V.; Shao-Horn, Y.; Itkis, D. M., Mechanism of Oxygen Reduction in Aprotic Li–Air Batteries: The Role of Carbon Electrode Surface Structure. *The Journal of Physical Chemistry C* **2017,** *121* (3), 1569-1577.
6. Kang, J.; Li, O. L.; Saito, N., Synthesis of structure-controlled carbon nano spheres by solution plasma process. *Carbon* **2013,** *60* (0), 292-298.
7. Chokradjaroen, C.; Kato, S.; Fujiwara, K.; Watanabe, H.; Ishii, T.; Ishizaki, T., A comparative study of undoped, boron-doped, and boron/fluorine dual-doped carbon nanoparticles obtained via solution plasma as catalysts for the oxygen reduction reaction. *Sustainable Energy & Fuels* **2020**.
8. Hyun, K.; Saito, N., The solution plasma process for heteroatom-carbon nanosheets: the role of precursors. *Scientific Reports* **2017,** *7* (1), 3825.
9. Phan, P. Q.; Chae, S.; Pornaroontham, P.; Muta, Y.; Kim, K.; Wang, X.; Saito, N., In situ synthesis of copper nanoparticles encapsulated by nitrogen-doped graphene at room temperature via solution plasma. *RSC Advances* **2020,** *10* (60), 36627-36635.
